# Mechanical Characterization in Red Blood Cells Using Optical Tweezers: A Review

**DOI:** 10.3390/bios16070379

**Published:** 2026-07-10

**Authors:** Xinyu Yang, Yuting Sun, Hong Jin, Jianguo Feng, Shangzhong Jin

**Affiliations:** 1 College of Optical and Electronic Technology, China Jiliang University, Hangzhou 310018, China; yangxy@cjlu.edu.cn; 2 Zhejiang Cancer Hospital, Hangzhou Institute of Medicine (HIM), Chinese Academy of Sciences, Hangzhou 310022, China; fengjg@zjcc.org.cn; 3 Sir Run Run Shaw Hospital, Zhejiang University School of Medicine, Hangzhou 310016, China; jinhong_512@163.com

**Keywords:** optical tweezers, red blood cells, mechanical properties

## Abstract

Given that red blood cells (RBCs) are the most abundant cells in blood, their morphology and mechanics strongly affect blood rheology. Furthermore, changes in the physiological functions and health status of an organism can also affect RBC mechanics. Therefore, understanding the mechanical properties of RBCs holds substantial research value in the biomedical field. The technology of optical tweezers (OT) has become a crucial method for measuring and analyzing the mechanical properties of RBCs, owing to their unique advantages such as non-contact manipulation and piconewton-level force sensitivity. This review first outlines the basic mechanical properties of RBCs, the mechanical sensing principles of optical tweezers, and their basic manipulation modes. It also focuses on the measurement and application of key mechanical parameters, such as the deformation index and shear modulus. Furthermore, the review covers the integration of optical tweezers with Raman spectroscopy, fluorescence, and microfluidics. These combined approaches allow for the simultaneous acquisition of mechanical and molecular data, dynamic monitoring of mechanical state changes, and analysis of external stimuli and physiological mechanisms, thereby supporting disease diagnosis, drug efficacy evaluation, and artificial blood quality assessment. Finally, it discusses current challenges and future directions.

## 1. Introduction

Red blood cells (RBCs), the most abundant cells in human blood, circulate throughout the vascular system and interact with diverse tissues. Therefore, their physical and biochemical properties are essential indicators for biomedical analysis and clinical diagnosis, with mechanical properties serving as key parameters reflecting cellular physiological function and pathological state. For example, high deformability enables RBCs to pass smoothly through capillaries narrower than 3 μm [1].

A number of studies have shown that abnormalities in RBC mechanical properties are closely associated with various diseases. For example, malaria infection leads to a significant increase in RBC membrane stiffness [2,3,4]; sickle cell anemia causes a substantial reduction in RBC deformability [5]; and RBC stiffness increases in diabetic patients [6]. This indicates that pathological conditions can significantly alter RBC mechanical properties, including deformability, shape recovery ability, elasticity, stiffness, and aggregability. Conversely, changes in these mechanical properties may serve as potential pathological markers. Because these markers provide mechanical information that cannot be replaced by conventional indicators, offering diagnostic and monitoring value, achieving accurate and quantitative measurements of RBC mechanical parameters has become an important direction in biomedical research.

Current techniques for characterizing the mechanical properties of RBCs can be broadly divided into population-based and single-cell measurements. Population-based techniques include microporous filtration, laser diffraction, microfluidics, and dielectrophoresis, while single-cell techniques include micropipette aspiration, atomic force microscopy (AFM), microfluidics, Brillouin microscopy, magnetic twisting cytometry, and optical tweezers (OT). Each technique has its own advantages and limitations, which are summarized in Table 1. And detailed principles can be found in the reviews performed by Matthews et al. [7], Liang et al. [8], and Xing et al. [9]. In addition, the force accuracy of techniques, which can directly measure mechanical values is also compared in Table 1. Among these, OT enable non-invasive, contact-free, sub-piconewton-force manipulation and quantitative measurement of stretching, rotation, and intercellular interaction forces. Due to their flexibility, high precision, minimal photothermal damage and extremely high force sensitivity, OT have become powerful tools to reveal the microscopic mechanisms governing RBC mechanical behavior.

**Figure 1:**
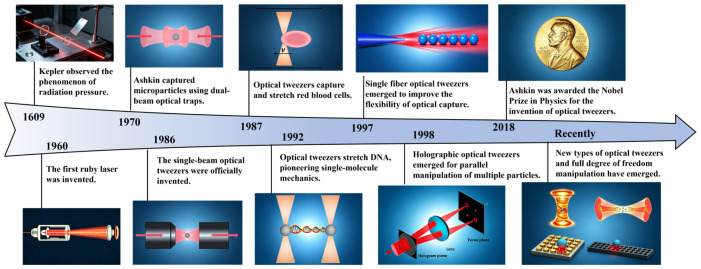
The evolution of optical tweezers since the pioneering work.

Figure 1 outlines the main development history of OT from the 1600s to today. Recently, the number of studies using OT to investigate RBCs has grown rapidly, and several reviews have discussed the research of OT on RBCs. For instance, the review written by Zhu et al. [10] provides a detailed introduction to the background and working principles of OT, as well as their various applications in RBC studies. However, it lacks a description of the manipulation modes of RBCs using OT and an introduction to the calculation formula of mechanical parameters. Although the work by Xie et al. [11] introduces different manipulation modes for RBCs by OT, including controlled deformation, dynamic stretching, aggregation–disaggregation behavior, and blood cell separation, it does not cover the measurement and calculation of mechanical parameters. On the other hand, the review performed by Liang et al. [8] focuses on the analysis of RBC deformability in various disease states through different techniques. However, it does not provide a detailed introduction to OT specifically. Furthermore, it does not discuss how the integration of multiple techniques can be applied to study RBC mechanical properties.

However, as attention to OT grows in RBC research, a systematic introduction to the field is still lacking. This review provides a technical and application-focused reference centered on RBCs and OT. It systematically describes the parameters used to characterize RBC mechanical properties and the working principles of OT. Then, it highlights two core manipulation modes (direct and indirect manipulation) along with sample preparation methods and the formulas for calculating mechanical parameters for each approach. The review also discusses how OT, combined with other techniques, can assess RBC mechanical properties under physiological and pathological conditions. In addition, it covers the role of OT in basic research on RBC aggregation and environmental stimuli, as well as the clinical significance of OT in blood storage, malaria, anemia, diabetes, and so on. Finally, the shortcomings and future development directions of OT in the study of RBC mechanical properties are pointed out. This work is expected to provide guidance for readers who have just entered the field.

**Table 1:** Summary of measurement contents, accuracy of force, advantages, limitations, and challenges of various techniques for measuring mechanical properties of red blood cells.

Technique	Measurements	Accuracy of Force	Main Advantages	Limitations and Challenges	References
Micropore filtration	Deformability.	Not providing the absolute value of force.	Simple operation; capability for batch measurements.	Low sensitivity; unable to quantitatively measure information from diseased subpopulations of RBCs.	[7,12,13]
Laser diffractometry	Deformability.	Not providing the absolute value of force.	Capability for batch measurements; rapid and simple; not affected by RBC aggregates or cell size variations.	Only measures population-average deformability, thereby losing quantitative information about diseased subpopulations of RBCs.	[14,15,16]
Microfluidic techniques	Deformability; relaxation time.	Indirectly calculate the force value based on the model, but the accuracy is affected by the model.	High throughput; small footprint; low sample consumption; supports both population-based and single-cell measurements.	Requires precise image processing; no access to RBC force data; needs integration, portability, and improved fabrication techniques.	[17,18,19]
Dielectrophoresis	Deformability; relaxation time; membrane shear modulus.	Indirectly calculate the force value based on the model, but the accuracy is affected by the model.	Integrable with microfluidics; relatively high throughput; label-free; no physical contact.	Difficult to precisely calibrate force magnitude and distribution; results affected by cell electrical properties; lacks a universal or clear mechanical model.	[20,21,22]
Micropipette aspiration	Membrane shear modulus; membrane bending modulus.	On the order of 10 pN [23].	Accurate measurement of individual RBC membrane mechanical parameters.	Time-consuming; low throughput; requires specialized equipment and trained personnel; potential cell damage during deformation.	[23,24,25]
Atomic force microscopy	Young’s modulus; adhesion force; membrane topography imaging.	On the order of 1 pN [26].	Extremely high sensitivity; precise measurement of individual cell membrane mechanical parameters.	Low throughput; only provides local membrane mechanical information; requires automation.	[9,27,28]
Brillouin microscopy	Elastic modulus.	Not providing the absolute value of force.	Non-contact; label-free; high-resolution 3D elastic modulus imaging.	High requirements for sample preparation; expensive equipment; complicated equipment adjustment; weak signal.	[29,30,31]
Magnetic tweezers	Dynamic modulus (membrane stiffness and loss modulus).	On the order of 1 pN [32].	High throughput; minimal photothermal damage.	Limited operational flexibility; non-uniform magnetic field and stress distribution; complex sample preparation.	[8,33,34]
Optical tweezers	Deformability; relaxation time; membrane shear modulus; elastic modulus.	On the order of 1 fN to sub-pN [35].	Extremely high force sensitivity; single-cell sorting and measurement; non-contact; label-free.	Requires specialized equipment and trained personnel; risk of photodamage; low throughput.	[10,36,37]

## 2. Manipulation of Red Blood Cells by Optical Tweezers

### 2.1. Mechanical Properties of Red Blood Cells

The unique mechanical properties of RBCs primarily stem from their distinctive structure and composition. Mature RBCs exhibit a biconcave disk shape, with a diameter of approximately 6~8 μm, a peripheral thickness of 2~2.5 μm, and a central thickness of 0.8 μm. Their primary structure comprises three components: an outer phospholipid bilayer membrane, a submembrane hexagonal scaffold network of ankyrin–actin, and an internal high-concentration hemoglobin solution [38]. These three components work together to maintain the morphological stability of RBCs and determine their mechanical behavior.

The mechanical properties of RBCs can be quantitatively characterized through three types of parameters: macroscopic, membrane microscopic, and rheological.

1. Among the macro parameters, the deformation degree of RBCs can be characterized by the elongation index (EI), relative elongation ratio (*ε*), deformation index (DI), and deformation ratio (DR), while the overall stiffness of RBCs was characterized by Young’s modulus (E). The following equations are commonly used.

The ratio of stretched RBC length to initial length is defined as the elongation index EI (or the elongation ratio) [39,40]:

(1) $$\mathrm{EI}=\frac{L}{L_{0}}$$

Then, the ratio of elongation to initial length is the relative elongation ratio *ε*:

(2) $$\varepsilon =\frac{L-L_{0}}{L_{0}}$$

where *L*_0_ is the initial major axis or minor axis, and *L* is the major axis or minor axis after stretching. EI represents the ratio of the total length of a RBC after being stretched to its original length in a given direction, emphasizing the “overall proportion after elongation”. *ε* represents the ratio of the increase in length of the RBC to its original length, emphasizing the “incremental part of the deformation”. EI and *ε* are related by the equation *ε* = EI − 1. In some articles, *ε* is also called the deformation index [41]. However, to facilitate comparative discussions of results obtained using OT, it is hoped that the nomenclature for the parameter corresponding to this formula will be standardized in the future.

In addition, if a RBC is stretched into an ellipse, the DI can be obtained by the classical ellipse-fitting method [16]:

(3) $$\mathrm{DI}=\frac{L_{x}-L_{y}}{L_{x}+L_{y}}$$

where *L_x_* is the length of RBCs along the stretching direction, and *L_y_* is the length of RBCs in the vertical stretching direction. DI quantifies the extent to which a RBC is deformed into an elliptical shape, ranging from 0 to 1. DI = 0 indicates that the RBC remains circular, whereas DI = 1 represents an extremely elongated RBC. This equation is commonly used in micropipette aspiration technology; microfluidic technology, where the channel size is larger than the diameter of RBCs; and laser diffraction technology.

If the RBC is deformed into a parachute shape, or the channel size is smaller than the RBC diameter, it can be characterized by DR [42]:

(4) $$\mathrm{DR}=\frac{L_{x}}{L_{y}}$$

DR represents the ratio of the lengths of RBCs in two perpendicular directions, emphasizing the “anisotropy of the shape”.

These four parameters, which are used to characterize deformability, all describe changes in RBC dimensions and shape. However, RBCs exhibit different deformation behaviors depending on the deformation scenario, which in turn determines the choice of formulas and parameter ranges for quantifying the degree of deformation in the reported articles. In a sample chamber with no spatial restrictions, RBCs can be stretched into elliptical or dumbbell shapes, allowing the use of Equations (1)–(3) to characterize their deformation. In contrast, when stretched through a narrow channel, the cell membrane frequently contacts the channel walls, leading to a parachute-like shape, in which case Equation (4) is appropriate for calculating RBC deformability.

When a RBC is subjected to indentation, its overall stiffness is characterized by Young’s modulus (E), as derived from the Hertz model [43]:

(5) $$E=\frac{3}{4}\times \frac{1-\nu^{2}}{\sqrt{Rh^{3}}}\times F$$

where *F* is the applied force, *ν* is the Poisson’s ratio of the RBC, *R* is the radius of the spherical indenter, and *h* is the indentation depth. In addition, the shear modulus (*μ*) can be calculated from E using the formula [43]:

(6) $$\mu =\frac{E}{2(1+\nu )}$$

Young’s modulus reflects the combined contributions of the membrane skeleton, cytoplasmic viscosity, and intracellular fluid pressure [44]. It can be derived from force–distance curves. While atomic force microscopy is the main technique for measuring Young’s modulus of RBCs [28], the indentation method based on OT can also be used [3,45]. RBCs under pathological conditions often exhibit a higher Young’s modulus than healthy cells, such as diabetes [46], sickle cell disease [47], and malaria [3]. In addition, RBCs with distinct morphologies also differ in their Young’s moduli [45,48].

2. Among the microscopic parameters of the membrane, shear stress and bending stress are the primary external forces applied. The membrane shear modulus (*μ*) reflects the membrane’s resistance to shear deformation; for example, healthy RBCs exhibit a *μ* of 2.4~11.3 μN/m, which increases tenfold during malaria infection [4]. Membrane shear viscosity (*η_m_*) and relaxation constant (*τ*) are typically quantified through post-stretching relaxation processes [49,50,51]. Membrane bending modulus (B ≈ 1.6 × 10^−19^ N·m) is obtained through localized membrane bending operations, with higher values indicating greater resistance to bending of the lipid bilayer [52,53,54].
3. Rheological parameters reflect the flow behavior of RBCs interacting with the blood environment (such as plasma and other blood cells), including aggregation index, aggregation time, aggregation velocity, aggregation force, disaggregation force, electrophoretic mobility, and suspension viscosity contribution. Aggregation index serves as an indicator of RBC aggregation, typically observed using a rheometer. Cell manipulation techniques can acquire aggregation and disaggregation forces, while high-speed microscopy provides aggregation time and velocity. These parameters can assess blood viscosity and assist in determining thrombotic risk; for example, RBC aggregation force in systemic lupus erythematosus patients is nearly twice as high as that of healthy individuals [55,56].

RBC mechanical properties are sensitive to physiological and pathological changes in internal and external environments. Therefore, they have emerged as valuable biomarkers for biomedical detection and diagnosis.

### 2.2. Optical Tweezer Technology

Ashkin’s team pioneered the OT technique, achieving stable capture of microspheres using counter-propagating laser beams [57] and a single highly focused laser beam [58]. They formally named this technique “OT” for capturing, driving, and separating particles.

**Figure 2:**
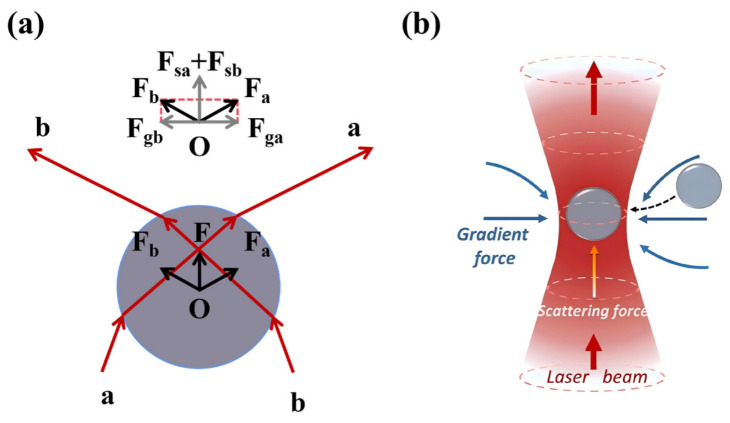
Principle of optical tweezers. (**a**) After penetrating a particle, the incident light undergoes a change in momentum direction, exerting a reaction force on the particle. F_ga_ and F_gb_ represent the gradient components of the optical force, while F_sa_ and F_sb_ denote the scattering components. Fa is the force exerted by light beam a on the particle, Fb is the force exerted by light beam b, and F is the combined force of both beams. (**b**) A focused Gaussian beam incident from below traps the particle at the focal point through the combined action of scattering and gradient forces. Adapted from Ref. [59] with permission.

When a Gaussian beam is focused, the light intensity near the focal point exhibits a three-dimensional gradient distribution. Due to momentum conservation, the incident light upon penetrating or refracting by the particle surface generates a scattering force (along the propagation) and a gradient force (along the intensity gradient) [58] (Figure 2a). Stable trapping occurs at the focal point where these forces balance. This condition is determined by optical parameters (wavelength, power, objective NA), particle properties (size, refractive index), and the surrounding medium. In the imaging path, integrated four-quadrant detectors enable sub-nanometer position tracking, transforming the trap into a piconewton-resolution mechanical sensor. For force calculation, Hooke’s law is typically used:

(7) F=k×Δx

where *k* is the optical trap stiffness, *∆x* is the distance between the center of the manipulated object and the center of the optical trap, and *F* is the optical trap force.

Hooke’s law is the principle underlying indirect force measurement in OT and is typically applied to trapping regular spherical objects. It requires calibration of trap stiffness via hydrodynamic or thermal motion analysis (reviewed in [35,60]), after which the force is calculated using Formula (7). For irregular objects (such as cells under direct manipulation), this approach may disrupt measurement stability.

Another indirect approach to obtaining optical trapping forces, *F_trap_*, employs Stokes’ law and is suitable for both regular and irregular objects (including RBCs). In practice, the trapped particle is subjected to a flow at a known velocity, and the resulting viscous drag is balanced by the trapping force at the point of escape. Thus, the trapping force can be computed based on hydrodynamic principles and Newton’s third law as [60]:

(8) $$F_{\mathrm{trap}}=-F_{\mathrm{Stokes}}=-6\pi \eta \mathrm{rv}$$

where *η* is the solution viscosity, *r* is the particle radius, and *v* is the flow velocity. With a constant increase in the relative velocity, the trapping force rises together. However, for RBCs, complex cellular fluid dynamics modeling is required to obtain accurate viscosity and optical trapping forces.

Recently, an optical momentum-based force sensing method has gained traction in cell-focused OT systems, requiring only an initial calibration after setup. This method directly computes the trapping force based on light-momentum changes. The optical trapping force can be directly calculated by [61]:

(9) $$F_{\mathrm{trap}}=\frac{R_{D}}{cf^{\prime }\psi }S$$

where *R_D_* denotes the effective dimension of the position-sensitive detector (PSD), *c* is the speed of light in vacuum, *f′* is the back focal length of the objective lens, *Ψ* is the PSD detection efficiency, and *S* is the PSD output electrical signal (converted from the collected optical momentum). These parameters do not depend on sample size, refractive index, or scattering characteristics, but they are only determined by the detection instrumental parameters.

In optical trapping applications, visible-wavelength lasers (such as 488 nm, 514.5 nm, or 532 nm) are frequently used for trapping non-biological particles. However, when applied to biological samples, these wavelengths tend to induce pronounced absorption, localized heating, and photodamage [62]. For this reason, near-infrared lasers (such as 970–1550 nm [63,64], especially at 1064 nm) are generally preferred for studies on biomaterials and living cells, as they significantly mitigate the risk of photodamage and have a lesser impact on RBC deformability and stiffness [65,66].

Based on differences in beam manipulation methods and system integration configurations, OT can be categorized into traditional optical tweezers, holographic optical tweezers (HOT), fiber-optic tweezers, surface plasmon optical tweezers, standing-wave optical tweezers, and photonic crystal optical tweezers [67,68]. This paper primarily focuses on traditional optical tweezers and holographic optical tweezers.

1. Traditional optical tweezers

**Figure 3:**
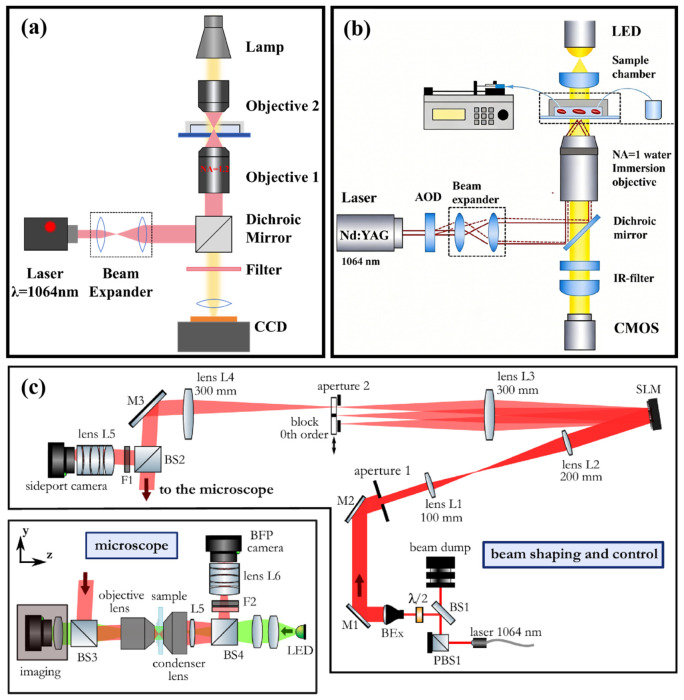
Schematic illustration of optical tweezer setups. (**a**) Conventional single-trap configuration. (**b**) Conventional dual-trap configuration implemented using an AOD. Adapted from Ref. [69] with permission. (**c**) Schematic illustration of a HOT setup. The system consists of two main components: beam-shaping control and an inverted microscope. Adapted from Ref. [75] with permission.

Traditional optical tweezers primarily comprise a near-infrared laser, beam expander, high numerical aperture (NA ≥ 1) oil or water immersion objective, and a three-dimensional nanoscale-translation stage. Among these, a high numerical aperture microscope objective and a single optical trap are the main characteristics of traditional setups. The expanded laser beam is focused onto the entrance pupil of the objective (Figure 3a), where the oil immersion medium reduces spherical aberration and forms a three-dimensional optical trap, with a focal-spot radius of ~0.6 λ/NA. Piezoelectric mirrors or PID-controlled displacement stages enable millisecond-scale nanoscale repositioning, supporting dragging, positioning, and stretching of RBCs. A beam-splitter prism at the objective’s back focal plane monitors transmitted light intensity. However, conventional systems generate only a single trap from one light source. Multiple optical trapping requires additional components, such as an acousto-optic deflector (AOD), for time multiplexing or a polarizing beam splitter (PBS) and scanning galvanometer for spatial multiplexing [60,69,70] (Figure 3b). Owing to switching-speed and diffraction-efficiency limitations, the number of traps remains restricted, and crosstalk between two closely positioned traps remains difficult to avoid [71].

2. Holographic optical tweezers

Inserting a spatial light modulator (SLM) before the laser enters the objective can effectively produce multiple dynamic optical traps that are parallel in both space and time [72,73] (Figure 3c). The SLM modulates the wavefront phase through a liquid-crystal pixel array, encoding the designed target dynamic optical traps into a phase hologram. The position of each focal spot is determined by the phase grating period, while the intensity is adjusted by the local phase depth. The spots can be spaced below the diffraction limit, which not only improves diffraction efficiency but also generates a dense trap array. Additionally, HOT are primarily used to grasp and drag non-spherical objects. With computer-generated holograms, tens of independent optical traps can be created to achieve the capture and parallel manipulation of both large non-spherical cells and multiple spherical particles [74]. Millisecond-level refresh rates enable real-time adjustment of these arrays, reducing experimental time and trapping errors. The ability to perform parallel, three-dimensional, and independent manipulation makes HOT ideal tools for single-cell mechanical characterization.

By applying external forces and monitoring real-time deformation, OT enable characterization of RBC mechanical responses under varying stress conditions and elucidation of associated structural mechanisms. Two primary manipulation approaches are employed: (1) direct manipulation, in which optical forces induce small cellular deformations; and (2) indirect manipulation, where microbeads attached to the RBC membrane are trapped and moved to deform cells.

### 2.3. Direct Manipulation

In direct manipulation studies, to maintain RBCs in suspension, RBCs are typically suspended in solutions including 0.9% sodium chloride solution, 0.01 M phosphate-buffered saline free of Mg^2+^ and Ca^2+^, and plasma [25,66,76]. Following suspension, sample chambers are frequently pre-treated with bovine serum albumin (BSA) (applied and air-dried) [76,77] or other agents, such as polylysine and casein [78,79], to prevent adhesion and ensure reliable measurements.

Under direct trapping by one or two optical traps, a RBC rotates from its flat-lying state to make its cell plane parallel to the optical axis. However, with three or more optical traps, the RBC remains stable lying flat. This phenomenon was first numerically validated by Tognato et al. using a ray-optics model of a native biconcave RBC [80]. Their findings indicate that, with one or two traps, optical torque drives RBC rotation, and the beam focus tends to stabilize at the thickest part of the cell; increasing the number of optical traps allows control over the RBC’s orientation.

To closely replicate RBC physiological conditions and acquire both the imaging and mechanical data during deformation, two direct optical manipulation methods are broadly applied: (1) single-tweezer trapping and dragging; (2) multi-tweezer stretching and manipulation.

#### 2.3.1. Trap-and-Drag Operation Based on a Single Optical Trap

**Figure 4:**
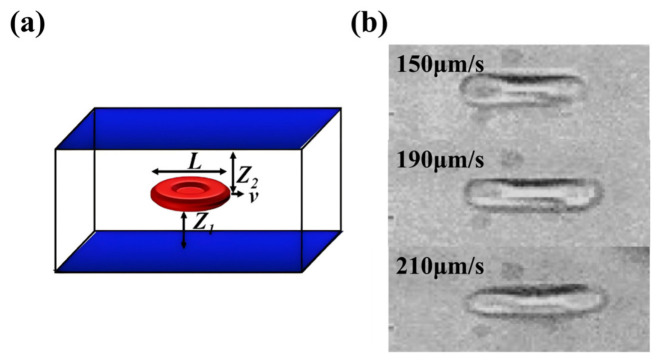
(**a**) Schematic diagram of single optical tweezers dragging a RBC, which is suspended in the sample chamber. Adapted from Ref. [66] with permission. (**b**) Microscopic image of RBCs dragged at different speeds. Adapted from Ref. [83] with Copyright 2011 IOP Publishing Ltd.

When one end of a RBC is held by an optical trap, its elasticity enables reversible deformation from a discocyte to a bullet-like shape under fluid shear stress (Figure 4). The membrane shear elasticity, *μ*, is calculated based on changes in the major axis [81] as:

(10) $$L=L_{0}+\frac{\eta L_{0}^{2}}{\mu Z_{eq}}\nu$$

with

(11) $$\frac{1}{Z_{eq}}=\frac{1}{Z_{1}}+\frac{1}{Z_{2}}$$

where *L* is the length of the RBC after deformation, and *L*_0_ is the length before deformation. *η* is the viscosity of the serum, plasma, or RBC suspension; *ν* is the velocity at which the optical trap drags the cell; *Z*_1_ is the distance from the RBC to the bottom of the sample chamber; and *Z*_2_ is the distance from the cell to the top of the chamber.

The shape recovery relaxation time, *τ*, is calculated from the exponential decay curve of the cell length [82] as:

(12) $$L=L_{0}+\Delta L\times e^{-\frac{t}{\tau }}$$

and the membrane viscosity, *η_m_*, is then determined [82] by:

(13) $$\eta_{m}=\tau \times \mu$$

Membrane shear elasticity, relaxation time, and membrane viscosity together characterize the ability and rate of RBCs to resist deformation.

Since it was proposed by Ashkin in 1987 [65], the single-beam OT “drag-and-deform” method has matured into a reproducible technique for quantifying RBC mechanical properties. To improve accuracy and reliability, a viscous drag-based calibration method based on the single trap was also developed to precisely measure optical trapping forces on RBCs [77]. Its applications span from measuring membrane elasticity and viscosity to assessing the effects of laser damage [66], storage lesions [81,84], and diseases like sickle cell anemia [5,85] and thalassemia [86]. Despite its established role in biophysical research, the method’s low experimental throughput remains a limitation for broader application.

#### 2.3.2. Stretch-and-Squeeze Operation Based on Dual Optical Tweezers and Multiple Optical Tweezers

**Figure 5:**
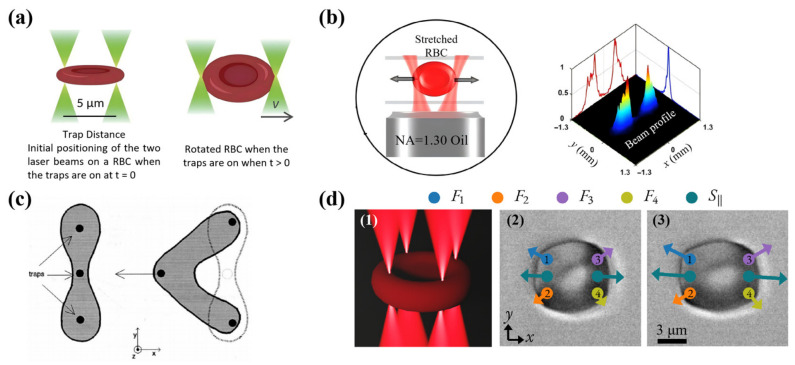
(**a**) Schematic of a RBC being trapped and stretched by dual optical tweezers. Adapted from Ref. [88] with permission. (**b**) “Tug-of-war” optical tweezer setup for stretching a RBC, along with the corresponding optical intensity distribution. Red and blue lines indicate the beam profiles in the x and y directions, respectively. Adapted from Ref. [77] with Copyright 2019 American Physical Society. (**c**) Bending of a RBC using three optical traps. HOT can also achieve this operation. Adapted from Ref. [49] with permission. (**d**) Application of HOT for the direct fixation and stretching of a RBC with four optical traps. (**1**) Schematic figure of a red blood cell held by four optical traps. (**2**,**3**) Image of the cell for the initial and maximally stretched states. Colored arrows indicate the applied forces and the effective stretching force S_‖_. Adapted from Ref. [75] with permission.

Another common method for probing RBC mechanics uses two optical traps—one stationary and one movable—to apply stretching force (Figure 5a). By measuring the cell’s initial length, maximum extension, and trapping force, key mechanical parameters can be derived, such as the deformation index [41,77,79], stiffness [48,76], shear modulus [77,87], and relaxation time [49,76].

To minimize photodamage, an innovative “tug-of-war” optical tweezer configuration, which generated divergent and elongated beams via SLM, was developed (Figure 5b). This system achieves a trapping force of 15 pN at just 20 mW, nearly double that of conventional dual Gaussian beam traps [77].

By varying the movement speed of the optical traps, it is possible to establish the correlation between DI and different cell morphologies, achieving cell classification [41,79,89]. In contrast to the macroscopic characterization provided by the deformation index, the shear modulus reflects microscopic membrane properties and is calculated from the measured deformation index and trapping forces. Recent studies have shown that *μ* decreases under hypotonic conditions due to increased surface area and reduced tension [77]. Furthermore, finite element modeling and analysis, performed with COMSOL v3.5 simulation software, enabled precise calculation of the stress distribution on the surface of deformed RBCs [87]. This work provided theoretical foundations for understanding the mechanical response of spherical RBC membranes in chemical environments.

Research on RBC mechanical dynamics has been advanced through new stretching modes and parameter definitions. Dual HOT enable stiffness measurement under small-strain conditions (<20%). Fatigue studies revealed that biconcave RBCs exhibit significantly greater stiffness increment (*Δk* = +2.3 pN/μm per cycle) than echinocytes during repeated stretching in early storage [48]. Here, the slope of the force-elongation curve under small-strain conditions is defined as the linear stiffness *k*, which is given by:

(14) $$k=\frac{dF}{dl}$$

where *F* is the optical trapping force and *l* is the RBC’s major axis length. Furthermore, successive stretch-relaxation cycles show a progressive linear decrease in DI, a power law increase in stiffness, and a 2.5-fold prolongation of relaxation time [76].

Although the two studies differ in how stiffness is defined, both approaches reveal consistent trends that capture the evolving elastic behavior of RBCs. Advancing beyond dual-trap systems, multi-trap configurations have enabled more complex RBC deformation studies. Three traps were used to form a “parachute-like” shape in RBCs, deriving an angular relaxation time that provides a unique metric for shape recovery dynamics [49] (Figure 5c). Recently, four holographic optical traps were also employed to symmetrically stretch RBCs while simultaneously monitoring deformation and forces in multiple directions [75] (Figure 5d).

Studies on RBC mechanical properties using multi-trap OT have collectively established a multidimensional framework for analyzing RBC mechanics, which offers key biomechanical indicators for applications in blood disease diagnostics, radiotherapy monitoring, and blood transfusion quality assessments.

### 2.4. Indirect Manipulation

Unlike direct optical stretching, the indirect method applies force non-invasively to minimize photodamage and avoid cell flipping. This maintains the RBC’s disk plane parallel to the focal plane, allowing CCD image acquisition and simultaneous analysis of both lateral and axial deformation.

Experimental sample preparation focuses on creating a “mechanical manipulation medium” by incubating RBCs with microbeads at a set ratio, forming stable attachments that act as “handles” for optical trapping. To prevent non-specific adhesion of cells or beads to chamber walls, which would interfere with measurements, BSA can be applied to coat the chamber interior or be added directly to the suspension [90,91,92].

In early stages, unmodified silica or polystyrene microbeads, with diameters ranging from 1~4 µm, were commonly used. Later, microbeads functionalized with IgG [93], carboxyl [94], streptavidin [95], or amino groups [92] were developed to enhance binding specificity and stability. Meanwhile, force calibration methodologies evolved from hydrodynamic flow to stage displacement, Boltzmann statistics, and thermal fluctuation analysis, improving measurement precision from the nanonewton to the piconewton scale [96].

**Figure 6:**
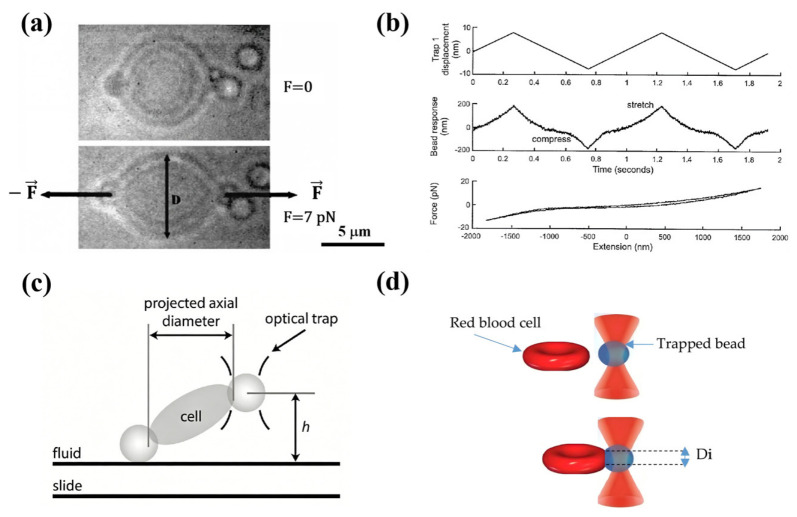
Methods of indirect manipulation of RBCs using optical tweezers. (**a**) RBCs are indirectly stretched using dual OT with microbeads. Adapted from Ref. [97] with permission. (**b**) Periodic triangular-wave or stepwise forces are applied to stretch RBCs. Adapted from Ref. [98] with permission. (**c**) One microbead attached to a RBC is fixed onto the glass surface, while the other is trapped by OT; stretching is achieved by moving the microscope stage. Adapted from Ref. [50] with permission. (**d**) Elastic stiffness of RBCs is measured using an indentation approach, where a microbead is pressed against the RBC surface. Adapted from Ref. [45] with permission.

The dual-bead attachment strategy was first introduced in 1999 with two operation modes: the “single-tweezer pulling and single-bead anchoring” mode and the “dual-tweezer trapping, anchoring and translation” mode [97]. By gradually increasing the distance between the two optical traps, a gradient tensile force was applied (Figure 6a). The shear modulus (*μ*) was derived from the relationship between the applied force and the change in cell diameter at the cell poles under small deformations, which is given by:

(15) $$L_{y}=L_{y0}-\frac{F}{2\pi \mu }$$

where *L_y_*_0_ is the initial lateral diameter of the RBC (vertical to the force direction), *L_y_* is the lateral diameter after deformation, and *F* is the applied tensile force. In the same year, Sleep et al. adopted a “fixed-translation” dual-tweezer configuration to investigate the contribution of the cytoskeletal network to the elasticity of nearly spherical RBCs [98]. Periodic triangular-wave or stepwise tensile forces were applied (Figure 6b), the parameters BH^2^ and shear modulus H were calculated based on Parker’s [99] axisymmetric shell elasticity theory:

(16) $$a{\mathrm{BH}}^{2}=(\frac{F}{5\varepsilon }{)}^{3}$$

where *a* is the bead radius, B is the bending modulus, *F* is the applied force, *ε* is the axial relative elongation ratio of the cell (see Equation (2)), and *L*_0_ and *L* are the initial and deformed axial diameters, respectively. Collectively, these two pioneering studies established a fundamental framework for calculating the RBC shear modulus, which has since become a reference standard for subsequent investigations. However, the spherical membrane model under small strain is different from the actual biconcave morphology of native cells.

Since 2003, OT stretching techniques have advanced toward large-deformation studies with improved precision. A finite element-based biconcave RBC model enabled accurate mechanical quantification, overcoming earlier spherical simplifications [100]. Subsequent refinements included bead height correction for reliable cell-length measurements and deformation data under high force ~193 pN [50] (Figure 6c). More recently, a microbead indentation method was developed to probe lateral membrane stiffness, yielding results consistent with micropipette aspiration while avoiding strong cell–substrate interactions [45] (Figure 6d).

With continuous advances in OT manipulation strategies, measurable parameters have expanded from the membrane shear modulus under small deformations [97] to a diverse set of multidimensional mechanical indicators, including the shear modulus under large deformations [100], relaxation time [50], intercellular adhesion forces [101], apparent membrane viscosity [101], stiffness [45], membrane fluctuation power spectral density [94], and dynamic changes in cell height during stretching [95].

## 3. Study of Red Blood Cells by Optical Tweezers Combined with Other Techniques

Although OT can measure the primary mechanical parameters of RBCs, additional data are often needed to clarify the underlying mechanisms. This has driven the integration of OT with complementary techniques, such as Raman microspectroscopy, fluorescence microspectroscopy, and microfluidics. Table 2 summarizes the parameter types accessible via individual techniques and their combined platform.

### 3.1. Optical Tweezers Combined with Raman Microspectroscopy

**Figure 7:**
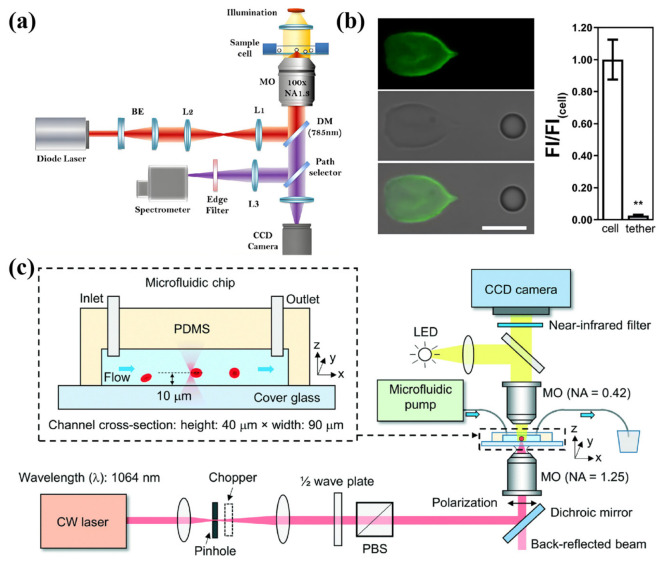
The combined application of optical tweezers with Raman spectroscopy technology, fluorescence spectroscopy technology, and microfluidic technology. (**a**) Schematic diagram of a setup where Raman spectral information from RBCs was simultaneously obtained during optical trapping. Adapted from Ref. [108] with permission. (**b**) A tether was pulled from a RBC using OT, after which the cell was chemically fixed and fluorescence stained. Imaging revealed no significant F-actin fluorescence signal in the tether region (** means *p* < 0.01 in *t*-test statistics, when comparing cell and tether experimental conditions). Adapted from Ref. [109] with Copyright 2020 American Physical Society. (**c**) Schematic diagram of the device for measuring the deformation rate of rabbit RBCs by an optofluidic “tweeze-and-drag” cell stretching system. Adapted from Ref. [110] with permission.

In conventional LTRS experiments, some laser beams function as optical traps to capture RBCs, apply mechanical forces, and induce deformation, while another beam is dedicated to Raman signal detection. Thus, LTRS has been used to simultaneously study the mechanical and chemical properties of RBCs (Figure 7a).

Using dual-trap stretching, an LTRS study revealed that β-thalassemia RBCs have a 40% higher membrane shear modulus and a weaker oxygenating capacity compared to normal RBCs [102]. These results indicate that the genetic defects underlying thalassemia, which primarily affect hemoglobin structure, also significantly impact RBC mechanical properties. Moreover, LTRS has been applied to investigate RBCs in malaria [103], sickle cell disease [104], and diabetes [105], highlighting its potential as a diagnostic tool.

However, a major limitation of LTRS is the difficulty in balancing Raman signal intensity with photothermal integrity. Since Raman scattering is intrinsically weak, signal enhancement usually demands higher laser power or longer collection times, which in turn increases photothermal damage to RBCs and affects their measured shear modulus.

To minimize laser-induced damage to RBCs in LTRS, a light-sheet Raman tweezer system that uses a single laser beam for both Raman excitation and optical trapping was developed. The system can achieve stable capture at a low power density of 3.8 × 10^4^ W/cm^2^, cutting power demand seven-fold [106] while enhancing biocompatibility and clinical potential by minimizing photodamage and heme aggregation.

On the other hand, Raj’s group [107] used an indirect stretching approach in which RBCs were tethered to microbeads, which significantly reduced laser-induced damage to the cells. At the same time, they found that once cell deformation exceeded 10%, several Raman peaks, corresponding to hemoglobin and protein vibration modes, rose and then saturated, suggesting that external force markedly alters the chemical structure of intracellular molecules.

With its “molecular fingerprint” characteristic, Raman spectroscopy enables non-destructive detection of structural changes in intracellular biomolecules. Its introduction endows OT with an additional capability about label-free chemical information detection.

### 3.2. Optical Tweezers Combined with Fluorescence Microspectroscopy

Conventional OT systems are incapable of three-dimensional imaging of RBCs, making it impossible to track the underlying causes of the shape changes observed from a restricted perspective (top-down view) after RBCs are captured. To overcome this limitation, Mohanty’s team [111] integrated confocal fluorescence microspectroscopy into an OT system, achieving fluorescence imaging and dynamic tracking of RBCs, revealing laser-induced orientation aligned with polarization rather than folding into rods.

Given that OT cannot discriminate between physical or chemical adhesion during RBC aggregation, flow cytometry was also combined with OT, demonstrating fibrinogen accumulation in cell-contact regions and confirming that glycoprotein receptor IIbIIIa (GPIIbIIIa)-mediated adhesion governs RBC aggregation, which can be reduced by GPIIbIIIa inhibitors [112].

To investigate the composition of RBC membrane tethers, researchers used a complementary setup of OT and confocal microscopy. Along with viscosity and bending modulus measurements, they showed that the membrane tether is mainly supported by the lipid bilayer, with no F-actin signal detected in tether regions [109] (Figure 7b).

Through specific labeling, fluorescence microscopy enables highly sensitive visualization of cellular structures, molecular distribution, and dynamic processes. The integration of OT with confocal fluorescence imaging and flow cytometry has progressively expanded RBC mechanics research from the overall elasticity of whole cells to morphological changes and mechanical regulatory mechanisms. However, a major difficulty lies in selecting appropriate fluorescent dyes, because the optical trapping laser can greatly accelerate their photobleaching. This causes a rapid decline in fluorescence signal and inevitably shortens the observation window for experiments.

### 3.3. Optical Tweezers Combined with Microfluidics

Conventional OT enables non-contact mechanical measurements but suffer from low throughput and difficulties in continuous analysis, whereas microfluidics offers high-throughput advantages but lacks precise single-cell manipulation. Combining high-precision optical manipulation with high-throughput microfluidics can break through the constraints of a single technology.

By integrating periodic chopped laser beams with microfluidic channels, researchers achieved non-contact stretching of RBCs during continuous transport [110] (Figure 7c). To overcome the low efficiency of single-channel capture and frequent clogging in multi-channel systems, a multi-channel microfluidic chip with pressure-release structures and time-shared OT was designed, achieving 100 cells/min throughput and >90% capture efficiency, and revealing membrane stiffening in pathological RBCs [69].

Beyond mechanical measurements, OT–microfluidic technology has also achieved label-free cell sorting. By exploiting differences in escape velocity, researchers obtained 95% sorting efficiency for healthy and thalassemic RBCs at a lower cost [113]. Another Artificial Intelligence (AI)-guided OT system can achieve automated, label-free separation of circulating tumor cells and RBCs in multi-channel microfluidics, with 100% separation purity [114].

The combination of OT and microfluidics overcomes their limitations, enabling efficient measurement of RBC mechanical properties and supporting clinical diagnosis. However, the feasibility of this method in complex and heterogeneous biological samples requires further validation. Moreover, microfluidic chip materials may introduce additional optical interference.

## 4. Application of Optical Tweezers in Red Blood Cell Research

### 4.1. Measurement and Study of Intercellular Interaction Forces

In physiological environments, RBCs usually do not exist in isolation; their functions rely heavily on intercellular interactions. The mechanical characteristics of RBC populations are directly reflected by their aggregation, disaggregation, and adhesion behaviors, which not only affect blood rheology and microcirculatory function but are also closely associated with various diseases. Therefore, the research scope of OT has extended beyond single-cell manipulation toward the investigation of collective mechanical properties of cell ensembles.

**Figure 8:**
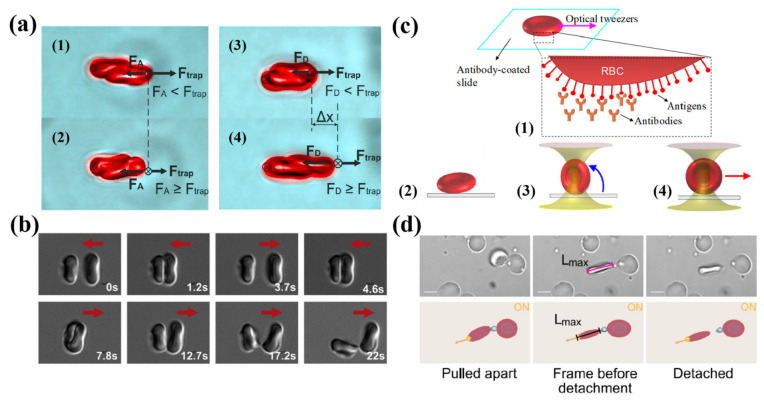
Recent multi-trap optical tweezer studies on RBC aggregation and adhesion. (**a**) Measurement of the aggregation force (F_A_) and disaggregation force (F_D_) of RBC doublets using dual optical traps. Adapted from Ref. [70] with permission. The minimum force preventing aggregation was defined as F_A_, while the constant force dragging the RBCs until the doublet separated was defined as F_D_. (**b**) Investigation of face-to-face adhesion and separation between two RBCs using four optical traps. During a recording period of 22 s, the cells were moved back and forth as indicated by the red arrows. Adapted from Ref. [115] with Copyright 2011 Elsevier Ltd. (**c**) Measurement of the binding force between RBCs and antibodies immobilized on glass substrates by pulling with OT; the RBC is lifted from the substrate, and the chamber is then moved at 5 μm/s and the trapping power reduced from 250 mW to 5~10 mW to evaluate RBC–antibody binding strength. (**1**,**2**,**3**,**4**) schematic illustration. Adapted from Ref. [116] with permission. (**d**) Measurement of the adhesion force between RBCs and malaria parasites using OT. Adapted from Ref. [117] with permission.

Using dual-trap and four-trap OT configurations to operate RBCs is a classical method for studying the aggregation characteristics of RBCs. Two RBCs were captured by dual traps and lifted 40 μm above the chamber bottom. The aggregation and disaggregation forces of RBC doublets were then measured by adjusting the light power and pulling dual optical traps (Figure 8a) [70]. This direct manipulation method can also be realized with only a set of double optical traps [83], which not only simplifies the operation steps, but also reduces the light damage of RBCs.

However, the traditional methods only focus on the aggregation and disaggregation behavior of RBC dimers under shear tension, ignoring the influence of the adhesive force between RBC disks on the aggregation behavior. A more physiological adhesion model was proposed by researchers, where four traps generated by HOT were positioned pairwise on two RBCs to apply forces perpendicular to the disk plane, simulating “collision and separation” under full contact (Figure 8b) [115]. Through the evaluation of the aggregation and disaggregation of RBCs by OT, the regulatory effects of laser radiation, nanoparticles, the life span of RBCs, adhesion factors and other factors on the interaction of RBCs have been explored [70,112,118,119].

These studies have advanced quantitative measurements of RBC interaction forces from various perspectives. However, most are in vitro experiments that involve extensive dilution, washing, and resuspension. Although beneficial for single-cell manipulation and mechanical measurements, such treatments do not replicate the shear forces, complex plasma composition, and multicomponent interactions found in real blood flow. Thus, current results are more appropriate as methodological validation.

Due to antigen variant recognition technology can promote early disease diagnosis, quantitative analysis of cell surface antigen variation has attracted more and more attention. Traditional flow cytometry requires a high number of samples, and cannot achieve quantitative analysis of a small number of samples and single cells. The cell-tearing operation based on OT can solve this problem (Figure 8c) [116]. This approach enables highly sensitive quantitative analysis only with minimal fingertip blood volume, offering a novel approach for detecting surface antigen variations. In addition, the interaction behavior and related mechanisms between RBCs and endothelial cells were explored by the same means [120].

These two studies have extended OT applications in RBC interaction research, showing strong methodological innovation. However, limitations remain: (1) using laser power as a substitute for absolute force simplifies experiments but reduces comparability with other mechanical studies; and (2) the change in RBC–endothelial adhesion force was weak and not statistically supported in all experimental groups.

In addition, OT can also quantitatively study RBC–merozoite adhesion forces through a triple-cell system where the merozoite acts as an adhesive bridge between two RBCs (Figure 8d). The traditional viewpoint holds that PfMSP1 protein dominates the initial attachment of Plasmodium, but high-precision OT experiments showed no significant change in adhesion forces after PfMSP1 knockdown [117]. In the Dantu blood group, it was also found that RBCs resist merozoite invasion not by reducing adhesion but by increasing membrane tension, providing key mechanical evidence for the screening of vaccine targets for malaria [78].

The samples in these two studies are representative, and cross-validation using multiple biological techniques makes their conclusions mechanistically convincing. However, such representativeness also introduces single-cell heterogeneity, requiring a large sample size to ensure data stability.

### 4.2. Study on Bioinformation of Red Blood Cells

The mechanical parameters of RBCs directly reflect membrane structural characteristics, physiological status, and species adaptability. With integration of biochemical analysis methods, OT have advanced RBC bioinformation research from qualitative mechanism exploration to quantitative characterization.

**Figure 9:**
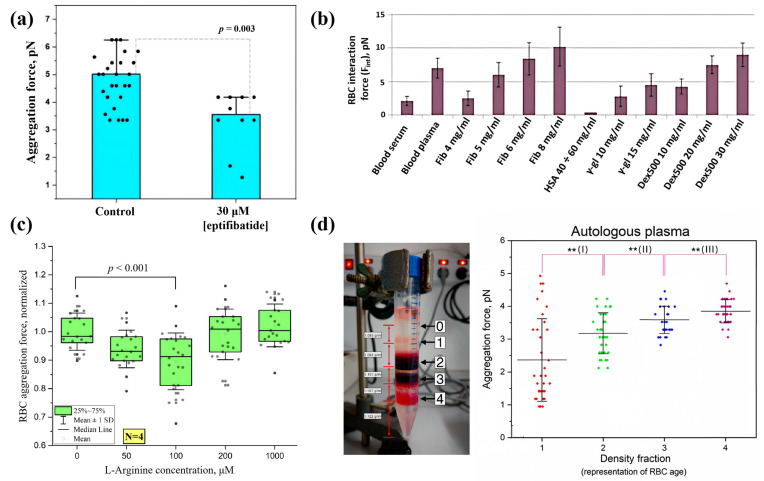
Studies on RBC bio-related information using optical tweezers. (**a**) A multiple optical trapping was used to investigate the effect of a GPIIbIIIa inhibitor on RBC aggregation properties. The results showed that the inhibitor significantly reduced the aggregation force. Adapted from Ref. [112] with permission. (**b**) Dual optical tweezers were employed to investigate the differential regulation of aggregation by various molecules, including autologous serum, autologous plasma, and phosphate-buffered saline solutions containing various macromolecules—Fib, fibrinogen; HSA, human serum albumin; γ-gl, γ-globulin from human serum; Dex500, 500 kDa dextran. Adapted from Ref. [16] with permission. (**c**) The influence of L-arginine concentration on RBC aggregation was quantitatively measured by OT. Adapted from Ref. [120] with permission. (**d**) Using multiple optical trapping, the aggregation forces of RBCs with different lifespans (corresponding to density fractions) were determined—where the youngest cells were positioned at the top and the oldest at the bottom. In autologous serum, the aggregation force increased progressively with cell age. In *t*-test statistics, ** (I) *p* = 0.003, ** (II) *p* = 0.002, ** (III) *p* = 0.004. Adapted from Ref. [118] with permission.

This technology has enabled quantitative analysis of key determinants in RBC aggregation. It was demonstrated that Ca^2+^ influx and phosphatidylserine exposure, rather than lysophosphatidic acid, are responsible for irreversible adhesion, excluding group-effect interference [115]. Using OT combined with laser aggregation assays and flow cytometry, researchers have shown that inhibition of GPIIbIIIa significantly reduced aggregation forces (Figure 9a), revealing a protein-dependent regulatory mechanism [112]. Subsequent studies further clarified the distinct roles of plasma proteins such as fibrinogen and albumin in modulating aggregation [16] (Figure 9b). Additionally, experiments demonstrated that nitric oxide regulates RBC–endothelial adhesion via the soluble guanylate cyclase (sGC) pathway, exhibiting a bell-shaped dependence on L-arginine concentration [120] (Figure 9c).

Although these studies included good negative and pharmacological controls (e.g., Ca^2+^ removal, GPIIbIIIa inhibitors, NO donors, or sGC inhibitors), some mechanisms remain largely inferred from pharmacological inhibitors, lacking direct molecular evidence to confirm specific receptors or binding sites.

RBCs exhibit varying lifespans within blood vessels, and experiments have shown that aggregation force increases with cell age [118] (Figure 9d), as well as that RBCs of varying densities exhibit significant differences in shear modulus [92]. This suggests that density-gradient centrifugation can effectively minimize sample heterogeneity and reduces measurement errors. However, Percoll gradient separation reflects RBC density rather than precise cell age. Thus, the classification of “young” versus “aged” RBCs requires further validation.

Additionally, the marked differences in DI and Raman spectral also revealed the differences between various morphologies of RBCs [79] and different species [89], such as camel (0.024 ± 0.019) and human (0.215 ± 0.061), offering new insights into the adaptive mechanisms of RBCs in extreme environments.

### 4.3. Study on the Effect of Different External Environmental Stimuli on Red Blood Cells

Investigating RBC intrinsic mechanical properties under complex microenvironments enhances understanding of relationships between their structure and function.

Laser irradiation studies demonstrated that wavelength and exposure dose modulate RBC stiffness and deformability, where 785 nm near-infrared light induces greater stiffening than 1064 nm [66], and visible-light irradiation triggers oxidative damage through photodissociation of hemoglobin-bound oxygen [121]. Continuous low-dose 450 nm exposure enhances deformability, whereas short-pulse He-Ne irradiation reduces aggregation force [70,122]. In radiobiology, high-dose irradiation decreases DI, making it a potential biomarker for radiation responses [88].

**Figure 10:**
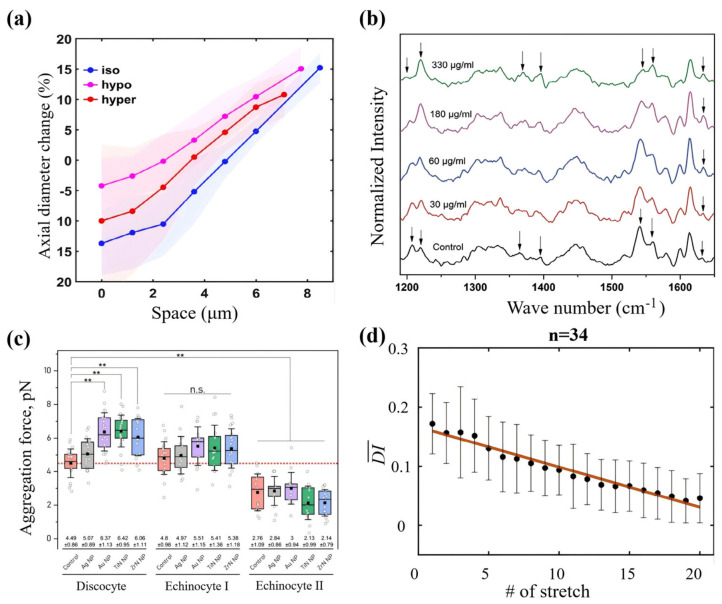
Studies on the effects of external stimuli on RBC mechanics using optical tweezers. (**a**) Dual optical traps were employed to directly stretch and compress RBCs, enabling analysis of their deformability in hypotonic (hypo), isotonic (iso), and hypertonic (hyper) solutions. Adapted from Ref. [77] with Copyright 2019 American Physical Society. (**b**) LTRS was used to study how bisphenol A with different concentrations affects Raman peaks associated with hemoglobin. The peak changes related to oxygenation markers are highlighted by arrows. Adapted from Ref. [124] with permission. (**c**) OT were used to investigate changes in RBC aggregation force after co-culturing with various nanoparticles, including Ag (silver), Au (gold), TiN (titanium nitride), ZrN (zirconium nitride), and NPs (nanoparticles). ** means *p* < 0.05, ns: non-significant, followed by Tukey’s test. Adapted from Ref. [119] with permission. (**d**) By repeatedly stretching RBCs with OT, the DI of the mechanical fatigue response was obtained. # means “number”. Adapted from Ref. [76] with permission.

Osmotic stress also plays a critical role in influencing RBC mechanical behavior. OT-based stretching and indentation experiments have shown that shear modulus and stiffness of RBC increase proportionally with osmotic pressure [45,93]. The deformability ranking is as follows: hypotonic > isotonic > hypertonic conditions [77] (Figure 10a). The study of LTRS at the molecular level revealed that osmotic changes alter the concentration of intracellular heme, reorganize membrane structures, and induce protein conformational variations, thereby regulating overall mechanical responses of RBCs [123].

Regarding chemical stimulation, LTRS demonstrated that bisphenol A damages membrane and hemoglobin structures [124] (Figure 10b); ethanol exposure induces deoxygenation and irreversible morphological loss [91]; and hydrogen peroxide increases shear modulus while reducing deformability [90], offering insights into environmental toxicology and blood-disease mechanisms.

With expanding biomedical applications of nanomaterials, nanoparticle (NP) biocompatibility with RBCs has drawn increasing attention. LTRS has shown that nanoparticles, including gold and silver, induce mechanical alterations in RBC membranes [108] (Figure 10c). Polymeric NPs exhibit weak interference with RBC interactions [125], while nitride-based plasmonic NPs have little effect on RBC deformability [119]. Therefore, these two types of NPs exhibit excellent biocompatibility, guiding the design of nanodrug carriers.

In mechanical fatigue studies, RBCs of different morphologies were repeatedly stretched using dual HOT, demonstrating that storage duration and stretching cycles increase stiffness and prolong recovery time (Figure 10d). These findings validated universal principles of “stretch-induced stiffness enhancement” and “rheological non-scalability”, revealing that accumulated mechanical strain drives rheological degradation of RBCs [48,76].

These studies have revealed RBC functional changes under external stimuli at multiple levels, including single-cell mechanics, morphology, and hemoglobin molecular state. However, most mechanistic interpretations rely on inferences from mechanical characterization, Raman shifts, or morphology, lacking direct validation of membrane skeleton proteins, ATP levels, oxidative damage markers, hemoglobin oxygen affinity, and ion channel function.

### 4.4. Study on Related Hematological Diseases

Hematological diseases are caused by abnormalities in blood cells, plasma proteins, or hematopoietic functions. OT and their combined technologies have been widely applied to the study of diseases related to RBCs, such as thalassemia [86,113,126] and sickle cell disease (SCD) [5,51,85].

Thalassemia induces abnormal mechanical and molecular properties of RBCs. LTRS has revealed that RBCs from β-thalassemia patients have significantly lower oxidative capacity and 40% higher stiffness than normal RBCs [102,126]. Additionally, RBC elasticity and membrane viscosity have shown decreased elasticity and increased membrane viscosity in diseased cells. Combined with quantum dot detection, a reduction in the negative surface charge of pathological RBC membranes has also been observed [86]. These findings suggest that disease-induced damage to membrane structures (such as surface glycoproteins and the cytoskeleton-associated band 3 protein) alters both the mechanical and electrical properties of RBCs.

In sickle cell disease (SCD), the HbS mutation leads to increased RBC rigidity and reduced deformability. Compared to healthy RBCs, the elastic modulus of RBCs from sickle cell trait (HbAS) donors significantly increased during storage, while hydroxyurea (HU) treatment restored deformability of RBCs [5,85]. In addition to measuring elastic modulus, 1064 nm optical traps allow non-destructive measurement of folding angle, rotational features, and deformation relaxation time of RBCs with SCD, showing more obvious distinctions in HU-treated samples [51,127]. The Raman tweezer systems also revealed that RBCs with SCD are more prone to deoxygenation under mechanical stress [104]. In addition, mannose-binding lectin (MBL), by binding to the membrane, alters both the membrane and the cytoskeleton, thereby increasing membrane stability and reducing the elasticity of RBCs with sickle cell disease (HbSS) [128]. This reveals the regulatory role of the immune molecule MBL in RBC mechanical properties.

However, the number of mechanical investigations on other disorders remains relatively limited, like acute myeloid leukemia (AML) [129], paroxysmal nocturnal hemoglobinuria (PNH) [130], hereditary elliptocytosis (HE) [131], iron deficiency anemia (IDA) [132].

### 4.5. Study on Red Blood Cells in Non-Hematological Diseases

Beyond hematological disorders, diseases such as malaria, cardiovascular disease, and diabetes also profoundly affect the RBC microenvironment, leading to abnormalities in aggregation, deformability, and elasticity.

**Figure 11:**
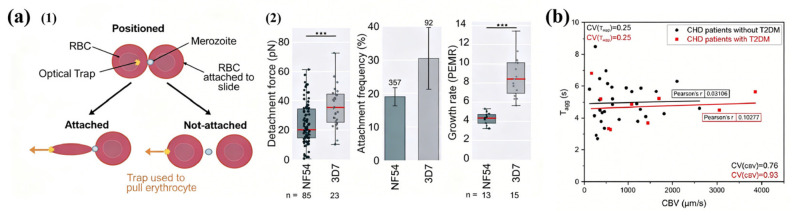
The mechanical properties of RBCs under non-hematological disease conditions. (**a**) OT were used to stretch a “RBC–Plasmodium–RBC” aggregate to explore detachment forces between merozoite-stage parasites lacking specific proteins and host RBCs. (**1**) This diagram shows the measurement method of attachment frequency and detachment forces. (**2**) These Box plots show the detachment force, attachment frequency, and growth rate between wild-type *P. falciparum* NF54 and 3D7 merozoites, respectively. *** means *p* ≤ 0.0007 in *t*-test statistics. Adapted from Ref. [117] with permission. (**b**) Aggregation time (T_agg_) of RBCs was examined for patients with coronary heart disease (CHD) and those with type 2 diabetes mellitus (T2DM). Adapted from Ref. [136] with permission.

During the malaria merozoite stage, the shear modulus of infected RBCs increases up to tenfold (≈53.3 N/m), indicating cytoskeletal remodeling by parasite-derived proteins [4]. Microsphere indentation experiments similarly revealed elevated shear stiffness [3]. Assuming stiffening of the local membrane skeleton, these experimental findings agree well with numerical calculations based on the Skalak constitutive model for RBCs, providing further insight into how *Plasmodium* infection affects the RBC membrane [133]. Crick’s group directly captured parasite invasion events and measured a detachment force of ~40 pN, further demonstrating that heparin significantly reduces adhesion strength and invasion rate [134]. Using OT combined with flicker spectroscopy, the Dantu blood group’s RBCs were shown to display higher membrane tension (1.22 × 10^−6^ N/m) and an invasion threshold of ~3.8 × 10^−7^ N/m, supporting the mechanism of high tension resistance against invasion [78]. Additionally, the combination of gene knockout and OT identified that the PfEBA/PfRH protein family, not PfMSP1, dominates adhesion, clarifying the molecular biomechanics of malaria invasion [117] (Figure 11a).

Cardiovascular diseases significantly modify RBCs rheology. In hypertensive patients, aggregation time decreased by 24% while the disaggregation force increased by 28%, providing one of the explanations for the elevated blood viscosity in hypertension, with the F_D_/F_A_ ratio showing diagnostic value [135]. Through combined OT–aggregometry–capillaroscopy analysis, aggregation time in CHD patients decreased by 27%, and those with CHD comorbid T2DM exhibited even stronger aggregation [136] (Figure 11b).

In diabetes mellitus, mechanical impairment of RBCs is strongly associated with microangiopathy. OT measurements indicated significantly reduced DI in type 2 diabetes and diabetic retinopathy, negatively correlated with initial RBCs size [41]. Fetal RBCs from gestational diabetes displayed increased membrane tension and bending modulus using a dual time-resolved membrane fluctuation spectroscopy based on OT [137]. Raman tweezers (785 nm) further detected distinct spectral differences at 1003 cm^−1^ and 753 cm^−1^, achieving 100% classification accuracy from diabetes erythrocyte using PCA-LDA [105]. Furthermore, OT technology provides a method for the early diagnosis of diabetes. Acute high-glucose exposure (early common symptoms of diabetes) enhanced oxidative stress, reduced membrane elasticity, and decreased elongation ratio with rising glucose concentration [138].

Table 3 compiles the alterations in RBC mechanical parameters observed across different disease disorders compared to healthy individuals. While OT remain limited to laboratory applications and lack depth exploration of the underlying molecular mechanisms, the mechanical data they generate may offer a theoretical foundation for the future design of cell mechanics-based diagnostic approaches.

### 4.6. Study on Drug Evaluation and Development

Drugs generally reach all parts of body through the blood circulatory system, which also affect mechanical properties of RBCs.

For pharmacological research, integrated with fluorescence imaging and flow cytometry, OT experiments revealed several drugs can reduce RBC membrane elasticity and stiffness, such as lithium salts, the first-line treatment for bipolar disorder [146]; vitamin E, an antioxidant used for stored RBC preservation [84]; and doxorubicin, a chemotherapeutic agent for acute myeloid leukemia [129]. These findings provide new biophysical evidence for understanding drug-induced cytotoxicity mechanisms.

Though Raman tweezers, common intravenous infusion solutions (such as 0.9% saline, Ringer’s lactate, and Plasmalyte-A) induced hemoglobin deoxygenation, impairing RBCs oxygen-carrying capacity, whereas RBCs in plasma demonstrated superior anti-deoxygenation properties [147,148]. Notably, hydroxyethyl starch, a solutions with safety concerns, was observed to impair both oxygenation state and membrane structure of RBCs [149].

These studies indicate that saline and other crystalloids should not be treated as “uninfluential backgrounds” when assessing drug effects on RBCs. Plasma or more physiological control conditions are recommended. Otherwise, drug and diluent effects may be confounded.

### 4.7. Application in Quality Evaluation of Artificial Red Blood Cells

Cultured RBCs (cRBCs) offer a promising solution to growing the demand for safe blood, yet their morphology and mechanical properties remain distinct from native RBCs (nRBCs), demanding high-precision evaluation.

The oscillatory OT system can employ an hourglass-shaped optical trap to capture cRBCs and apply axial oscillations. By analyzing high-frequency scattering signals and membrane fluctuations, results showed that cRBCs, cultured with human platelet lysate, exhibited biomechanical properties most similar to nRBCs, consistent with measurements from digital holographic microscopy [150]. Despite comparable membrane elasticity and protein expression, a lipid deficiency in cRBCs caused their morphological and mechanical impairments, which was analyzed by integrated OT-AFM-SEM. This finding highlights the utility of OT as critical tools for evaluating cRBC quality [44].

However, these conclusions are mainly based on in vitro single-cell experiments, and factors like maturation stage, cell heterogeneity, and measurement methods may influence the mechanical parameters. Thus, further validation (e.g., causal lipid interventions, high-resolution membrane skeleton analysis, and in vivo models) is needed to confirm whether these mechanical similarities can assess cRBC quality.

## 5. Conclusions and Outlook

OT have developed into critical tool in RBC mechanical research. Their evolution is marked by three key advances: the transition from basic manipulation to performance-optimized systems, the integration with complementary techniques for multidimensional analysis, and the expansion from studying healthy cells to the applications in disease diagnosis. These developments have revealed the relationship and differences between the global and local mechanical properties of RBCs, as well as their interaction mechanisms, providing new insights for medical diagnostics.

However, OT technology still faces several challenges in RBC mechanical research. (1) Current studies mainly focus on highly RBC-associated diseases (e.g., sickle cell disease, malaria, and diabetes), whereas research on diseases with lower correlation but potential value (e.g., Alzheimer’s disease) remains insufficient. Moreover, limited clinical samples also hinder the development of its clinical application due to the lack of a large number of standard reference values. (2) OT have been demonstrated to trap and manipulate RBCs in zebrafish caudal arteries and mouse ear capillaries [151,152]. However, due to multiple light scattering in tissue, a Gaussian beam cannot form an effective optical trap at depth, making in vivo manipulation in humans currently unachievable. (3) More critically, most OT systems are low throughput, making them unsuitable for rapid clinical testing and large-scale studies.

Looking ahead, the development directions of OT in RBC mechanical studies are becoming clear. Currently, most OT-based RBC studies focus on the study of ex vivo RBCs. The development of in vitro artificial RBC culturing technology is beneficial to combine OT with gene editing technology and membrane component analysis of controlled variables. It not only reduces the complexity of sample source information that affects experiments but also facilitates mechanism analysis. At the same time, in vivo OT not only provides a natural environment that cannot be reproduced by ex vivo RBCs but also promotes research on diagnostic applications. The problem of strong scattering of biological tissue can be solved through wavefront regulation by enhancing laser focusing [153]. These development directions provide a foundation for the clinical application of OT. In addition, a large amount of experimental data is still needed in this field to determine standard methods and diagnostic indicator ranges.

In addition, the future integration of OT with AI holds great potential for resolving efficiency challenges. Since the calculation of RBC mechanical parameters relies on morphological data, AI-powered image analysis can rapidly extract such data from images and videos in batch, drastically cutting processing time. This method is also well-suited for OT–microfluidic integration, as it supports higher detection throughput. In addition, deep learning-based models can perform feature analysis on multi-dimensional mechanical parameters, allowing multi-parameter feature extraction and highly sensitive discrimination. The selection of optical trap positions mostly requires manual input from experts, systems equipped with Machine-Guided Isolation of Cells (MaGIC) enable target cell capture and sorting in multiple channels without operators, facilitating automation. Research in this direction remains scarce but is highly promising. AI-driven OT research is expected to evolve the technology from that relies heavily on expert experience into an efficient and standardized tool for routine use.

In summary, OT allow for the multi-parametric characterization of RBC mechanical properties, which hold potential as physical biomarkers to support both basic RBC research and disease diagnosis. Nevertheless, significant challenges still exist with regard to in vivo applications, throughput, and standardization.

## Figures and Tables

**Table 2:** Summary of measurable parameters of optical tweezer technology and combination technology platform. LTRS: laser trapping with Raman spectroscopy.

	OT	Raman Microspectroscopy	LTRS	Fluorescence Microspectroscopy	OT & Fluorescence Microspectroscopy	Microfluidics	OT & Microfluidics
Diameter of RBCs	√	√	√	√	√	√	√
Deformation index	√	—	√	—	√	√	√
Force	√	—	√	—	√	—	√
Membrane shear modulus	√	—	√	—	√	√	√
Young’s modulus	√	—	√	—	√	√	√
Relaxation time	√	—	√	—	√	√	√
Raman signal	—	√	√	—	—	—	—
Fluorescence signal	—	—	—	√	√	—	—

“√” indicates that parameter is measurable; “—” indicates that parameter is not measurable.

**Table 3:** Mechanical characteristics of red blood cells in some disease states discovered through optical tweezers.

Disorder	Mechanical Property	Related Mechanisms	The Foundation of Auxiliary Diagnostic Indicators
Thalassemia	Less elasticity; higher membrane viscosity; longer relaxation time. Decreased oxygenation capability.	Abnormal globin deposits on the RBC membrane, impairing membrane structure and cation channel activity [139]. Abnormal associations among the α, β, and γ chains lead to increased oxygen affinity and decreased oxygen release capacity [140].	Membrane stiffness increases by 22.4% to 40% [86,102].
Sickle cell disease	Increased RBC rigidity; reduced deformability; longer relaxation time. Easy to deoxygenate.	β-globin mutation generates HbS, which aggregates upon deoxygenation, exacerbating oxygen affinity reduction and restricting cytoskeletal–membrane fluidity [141].	Membrane stiffness and elastic modulus increase by 40% to 44% [85,128].
Malaria	Increased membrane shear modulus; Young’s modulus, adhesion. and membrane tension; reduced deformability. Easy to deoxygenate.	Plasmodium proteins and invasion modify RBC membrane composition and cytoskeleton, while hemoglobin digestion releases H_2_O_2_ that causes oxidative damage [142].	With malaria parasite developing, membrane shear modulus increases by 1.5 to 10 times [4]. Young’s modulus increases by 10 times [3].
Hypertension	Reduced aggregation time; increased disaggregation force.	Bridging proteins (especially fibrinogen) used for aggregation are increased in the plasma of hypertensive patients, showing stronger aggregation [143].	Aggregation time reduced by 24%. Disaggregation force increased by 28% [135].
Coronary heart disease	Reduced aggregation time.	Elevated plasma macromolecular proteins (especially fibrinogen) and reduced membrane sialic acid levels diminish intercellular repulsion [144].	Aggregation time reduced by 27% [136].
Diabetes mellitus	Reduced deformability and elasticity; increased membrane tension and bending modulus. Abnormally increased and irreversible oxygen affinity.	Increased RBC stiffness: elevated reactive oxygen species and non-enzymatic glycation of membrane proteins. Increased hemoglobin oxygen affinity: higher glycated hemoglobin levels [145].	Deformation ability has decreased by 7.6% to 9% [41]. Membrane tension increased by 22.6%, and bending modulus increased to 2.29 times [137].

## Data Availability

No new data were created or analyzed in this study.

## Abbreviations

The following abbreviations are used in this manuscript:

RBC	Red blood cell
OT	Optical tweezers
AFM	Atomic force microscopy
EI	Elongation index
DI	Deformation index
DR	Deformation ratio
PSD	Position-sensitive detector
HOT	Holographic optical tweezers
AOD	Acousto-optic deflector
PBS	Polarizing beam splitter
SLM	Spatial light modulator
BSA	Bovine serum albumin
LTRS	Laser trapping with Raman spectroscopy
GPIIbIIIa	Glycoprotein receptor IIbIIIa
HSA	Human serum albumin
sGC	Soluble guanylate cyclase
NP	Nanoparticle
SCD	Sickle cell disease
Hbβ	Hemoglobin beta
HbA	Hemoglobin A
HbAS	Hemoglobin A-S
HU	Hydroxyurea
MBL	Mannose-binding lectin
HbSS	Hemoglobin S-S
AML	Acute myeloid leukemia
PNH	Paroxysmal nocturnal hemoglobinuria
HE	Hereditary elliptocytosis
CHD	Coronary heart disease
T2DM	Type 2 diabetes mellitus
cRBC	Cultured red blood cell
nRBC	Native red blood cell
